# Atomistic Detailed Mechanism and Weak Cation-Conducting Activity of HIV-1 Vpu Revealed by Free Energy Calculations

**DOI:** 10.1371/journal.pone.0112983

**Published:** 2014-11-13

**Authors:** Siladitya Padhi, Raghunadha Reddy Burri, Shahid Jameel, U. Deva Priyakumar

**Affiliations:** 1 Centre for Computational Natural Sciences and Bioinformatics, International Institute of Information Technology, Hyderabad, India; 2 International Centre for Genetic Engineering and Biotechnology, New Delhi, India; Jacobs University Bremen, Germany

## Abstract

The viral protein U (Vpu) encoded by HIV-1 has been shown to assist in the detachment of virion particles from infected cells. Vpu forms cation-specific ion channels in host cells, and has been proposed as a potential drug target. An understanding of the mechanism of ion transport through Vpu is desirable, but remains limited because of the unavailability of an experimental structure of the channel. Using a structure of the pentameric form of Vpu – modeled and validated based on available experimental data – umbrella sampling molecular dynamics simulations (cumulative simulation time of more than 0.4 µs) were employed to elucidate the energetics and the molecular mechanism of ion transport in Vpu. Free energy profiles corresponding to the permeation of Na^+^ and K^+^ were found to be similar to each other indicating lack of ion selection, consistent with previous experimental studies. The Ser23 residue is shown to enhance ion transport via two mechanisms: creating a weak binding site, and increasing the effective hydrophilic length of the channel, both of which have previously been hypothesized in experiments. A two-dimensional free energy landscape has been computed to model multiple ion permeation, based on which a mechanism for ion conduction is proposed. It is shown that only one ion can pass through the channel at a time. This, along with a stretch of hydrophobic residues in the transmembrane domain of Vpu, explains the slow kinetics of ion conduction. The results are consistent with previous conductance studies that showed Vpu to be a weakly conducting ion channel.

## Introduction

**Figure 1:**
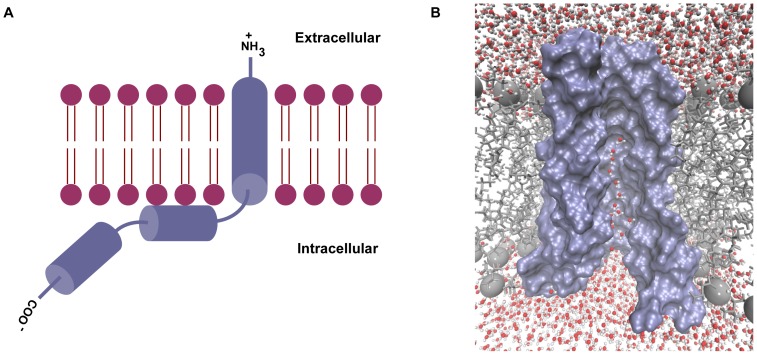
The structure of Vpu. (A) Schematic illustration of the monomer. (B). The pentameric channel set up in a hydrated lipid bilayer. The monomeric unit on the front has been omitted to reveal the interior of the pore. Pore water molecules can be seen in the pore lumen.

The viral protein U (Vpu) is one of the four accessory proteins encoded by HIV-1 that has an N-terminal transmembrane (TM) domain and a C-terminal cytoplasmic domain [1]–[10]. A schematic representation of the protein is shown in Figure 1 (A). The cytoplasmic domain has two α-helices [11], [12], and is involved in the degradation of CD4 molecules in the host cell [13], [14]. The TM domain, which is known to have a helical topology [15]–[20], has been suggested to enhance virus release via two mechanisms: formation of ion-conducting channels [21], [22] and degradation of the antiviral protein tetherin [23]. The ion channel is formed via oligomerization of monomeric Vpu to form a pentamer [24]–[26], [16]. Because of its importance in virus release, an understanding of the mechanism of ion permeation is of great importance. Vpu has been reported to show ion channel activity in lipid bilayers, *Xenopus* oocytes, and in the plasma membrane of *Escherichia coli* [21], [22]. Vpu channel has been shown to be selective towards monocations (Na^+^ and K^+^), and cannot differentiate between the two [21], [22].

Channel recordings on full length Vpu (Vpu_1–81_) and the Vpu TM region (Vpu_1–32_) excluding the cytoplasmic domain showed that the conductance states and ion-conduction recordings are similar for the two [27]. The channel has been suggested to exist in a number of conductance states, which are believed to correspond to different open state conformations. Furthermore, the duration for which the channel remains open is independent of the voltage, and the channel shows biphasic voltage activation. The variation of conductivity with respect to salt concentration has been measured by Mehnert et al., which showed that ion conduction follows Michaelis-Menten behavior [28]. The ion transport activity is rather weak, though the channel shows pore-like characteristics. A mutant Vpu with Ser23→Leu mutation in the TM domain does not conduct ions, suggesting a critical role for serine in ion transport [28]. Two possible roles have been ascribed to the serine residue. Firstly, it might act as a weak ion binding site, and secondly, it creates a hydrophilic environment in the vicinity, thereby reducing the length of the hydrophobic stretch that occurs inside the channel, thus making it easier for an ion to pass through. The passage of ions through the pore has been previously modeled using steered molecular dynamics (SMD) simulations [29]. The possibility of the existence of a number of conformations of oligomeric Vpu has recently been suggested by computational studies that have modeled the assembly of monomeric Vpu into oligomers [30]. This supports experimental channel recordings that suggested the existence of a number of conductance states of Vpu [27].

Molecular dynamics (MD) simulations have been successfully used to study transmembrane proteins in general [31]–[41]. A powerful approach is the use of umbrella sampling MD simulations, which are in general very helpful in understanding free energy changes corresponding to rare events [42]–[45]. This method has been used by Roux and coworkers for elucidating the energetics of ion permeation through channels [46]–[49]. This method also has been used to obtain a multi-ion free energy profile for the KcsA K^+^ channel [46]. Results from that study showed that an ion trapped in the selectivity filter is able to proceed further into the channel because of electrostatic repulsion from ions that subsequently approach the selectivity filter. Based on this, it was proposed that rapid conduction in the channel is driven by interionic repulsion. The method has subsequently been used for calculating the free energy profile of ion transport through the gramicidin channel as a function of both axial and radial positions [47], and for elucidating how the KcsA channel preferentially allows K^+^ rather than Na^+^ ions to pass through [49].

The present study employs umbrella sampling MD simulations to elucidate the mechanism of ion conduction through the Vpu channel. We have recently reported a molecular model for the pentameric state of the TM domain of Vpu protein, which has been used here [24]. Available experimental information on Vpu was used to validate the proposed structure. Since only the TM domain is involved in channel activity [22] and the conductance properties of channels formed by the Vpu TM region are almost the same as those formed by full length Vpu [27], the cytoplasmic domain of Vpu has not been included in the model. This study explains the molecular and energetic basis of ion selectivity in the channel, and suggests that a hydrophobic stretch in the channel might control the kinetics of the ion permeation process. Finally, a mechanism for the transport of ions is proposed based on a multi-ion free energy landscape.

## Methods

The Vpu channel structure used in the study is a pentameric form of the TM region modeled in a recent study [24]. In addition to the protein, the system includes 95 lipid (POPC) molecules, 5496 water molecules, 13 K^+^ ions and 18 Cl^−^ ions, with a total of 31984 atoms. Prior to the current study, the system was equilibrated for 30 ns without any restraints and was found to be adequately equilibrated, as evidenced from the convergence of RMSD, the occurrence of several stabilizing intra-protein and protein-lipid interactions, and the retaining of the structural integrity of the channel, in general.

The transport of two different ions through the channel was studied, namely Na^+^ and K^+^. For modeling the permeation of a K^+^ ion, the coordinates of a K^+^ ion from bulk water were first swapped with a water molecule at the C-terminus of the channel. Conformations with the K^+^ ion occurring at different positions along the axis of the pore were generated by pulling the ion along the pore axis using steered molecular dynamics (SMD) simulations. The ion was pulled at a constant velocity of 0.01 Å ps^−1^ by applying an external force on a dummy atom connected to the permeating ion via a spring with a harmonic constant of 10 kcal mol^−1^ Å^−2^. The structural model for the channel was aligned along the z-axis [24], and hence the pulling force was applied along the z-axis, thereby making the ion move from the C-terminus to the N-terminus. Positional restraints with force constant 1 kcal mol^−1^ Å^−2^ were applied on the heavy atoms of the protein to prevent the drifting of the helices during the pulling of the ion. Constant temperature and pressure were employed by using Langevin dynamics and a Langevin pressure piston, respectively. It must be noted that the SMD simulations were used to obtain the initial structures for the umbrella sampling simulations and not for investigating the permeation mechanism. The NAMD program [50], [51] was used for the SMD simulations with the CHARMM22 all-atom protein force field with CMAP corrections [52], [53], the CHARMM36 all-atom lipid force field [54], optimized parameters for ions [55], and the TIP3P water model [56]. For the studies on the permeation of Na^+^, the conformations were generated by replacing the K^+^ ion in the pore with an Na^+^ ion.

Umbrella sampling was performed by applying a biasing potential on the permeating ion, with the potential having a parabolic form. Independent simulations were performed for different positions of the ion along the pore axis, so that each simulation sampled the conformations of the system with the ion at the respective region. For a window *i*, the biasing potential has the form
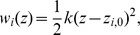
where *k* is the harmonic constant, *z_i,0_* is the center of the window *i*, and *z* is the position of the ion along the reaction coordinate at any given instant. The potential energy function then has the form
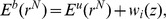
where r^N^ denotes the positions of all the atoms in the system, and E_u_(r^N^) is the unbiased potential energy function. The unbiased probability distribution of the ion along the pore axis *P_i_^u^(z)* is computed from the biased probability distribution *P_i_^b^(z)*, and is given by
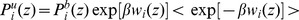
The free energy along the reaction coordinate is then given by
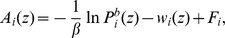
where *F_i_* is a constant for window *i*. There are several approaches for determining *F_i_*, of which the most popular is the weighted histogram analysis method (WHAM) [57]–[59]. It calculates the unbiased global distribution *P^u^(z)* using the relation
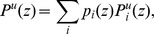
where *p_i_(z)* is the weight for window *i* which ensures that the statistical error for *P_u_(z)* is minimized. It is given by
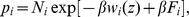
where *N_i_* is the number of steps sampled for window *i*. *F_i_* is calculated using the relation
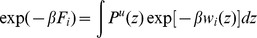

*P^u^(z)* and *F_i_* are calculated in a self-consistent manner using the above equations.

Conformations with the ion placed at different places along the pore axis were extracted from the SMD simulation, and were used as initial structures for the different windows in the umbrella sampling calculations. The ion position ranged from *z* = −30 Å to *z* = 36 Å, giving a total of 133 windows along the z-axis spaced 0.5 Å apart (the model structure used in the study had previously been aligned along the z-axis). A distribution of ion positions around the center of a given window was obtained by applying a biasing potential with a force constant of 20 kcal mol^−1^ Å^−2^ on the ion using the MMFP module in the CHARMM program [60]. Positional restraints with force constant 1 kcal^−1^ mol^−1^ Å^−2^ were applied on the backbone C_α_ atoms of the protein to prevent the drifting of the channel. Ions other than the permeating ion were excluded from the pore region by using a repulsive sphere of radius 15 Å, with the repulsive force acting on ions only when they entered this sphere. Covalent bonds involving hydrogen were constrained using SHAKE [61]. Simulations were carried out in the NPT ensemble using the CHARMM program [60] with the same force field parameters as those used in the pulling simulations mentioned above. Simulations for each window involved 100 ps of equilibration followed by 1 ns of production, with a simulation time of (133 * 1.1) ns for each free energy profile. For calculating the potential of mean force (PMF) for the transport of the ion across the channel, the umbrella sampling trajectories were unbiased using WHAM [57]–[59].

The permeation of two K^+^ ions through the channel was modeled by considering different positions of one ion relative to the second ion, with the spacing between the two ions varying from 4 Å to 10 Å (additional windows with 11 Å and 12 Å spacing between the ions were sampled for the middle region of the channel). A total of 395 windows spaced 1 Å apart were sampled with the two ions at different positions along the pore axis. A biasing harmonic potential of force constant 10 kcal mol^−1^ Å^−2^ was applied on the two ions. Parameters for the other restraints used were the same as those used for the calculations with a single ion in the pore. Equilibration and production runs for each window were carried out for 100 ps and 200 ps, respectively. The total simulation time combining all the single ion and two-ion simulations was more than 0.4 µs, with a total of 661 independent simulations. All molecular images were rendered using the visual molecular dynamics (VMD) program [62]. An electrostatic surface representation of the channel was generated using the PDB2PQR server [63].

## Results and Discussion

### Atomistic structure of TM domain of Vpu, and features of the lumen of the pore

**Figure 2:**
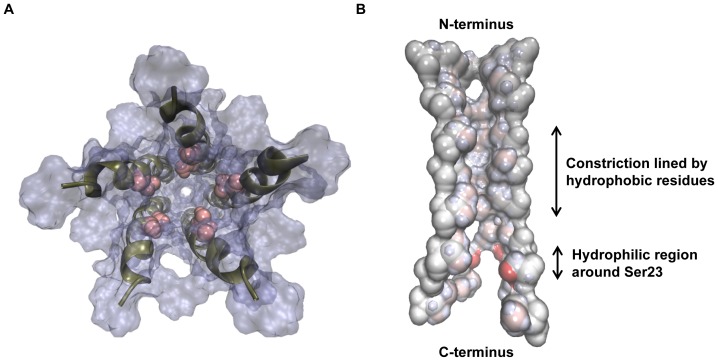
Electrostatic profile of pore-lining residues. (A) The pentamer model used in the study shown with the Ser23 residue in van der Waals representation. (B) A representation of the electrostatic surface of pore-lining residues in the channel. The monomer unit on the front has been omitted for clarity. Red indicates negatively charged regions, blue indicates positively charged regions, and white indicates nonpolar regions. Only the sidechain atoms are shown in color, and the backbone atoms are in white to allow a clear illustration of the nature of pore-lining residues.

A representation of the channel modeled in a hydrated lipid bilayer is shown in Figure 1 (B). A few pore water molecules can be seen, especially in the bottom half of the pore, towards the C-terminal opening. The channel consists of five helical TM domains held together predominantly by van der Waals forces, with the interface between the helices being formed by nonpolar residues [24]. The channel is wider at the C-terminal end than at the N-terminal end, leading to a relatively greater degree of hydration at this end. The residues Arg30 and Tyr29 at the C-terminal end of the TM domain face the exterior side of the channel, and they stabilize the protein in the lipid bilayer by forming hydrogen bonds with phospholipid headgroups [24]. The sidechain of Arg30 of each of the monomers is also able to form a salt bridge with a Glu28 residue on the adjacent monomer. Although Lys31 is directed towards the interior of the channel, it lies in a region that is sufficiently solvated, so that the residue is shielded from exerting any influence on any ion passing through the channel. The orientation of these residues in the channel is shown in Figure S1 (A). A serine at position 23 faces the lumen of the pore, and it marks the end of the small hydrophilic space at the C-terminal entrance of the channel. Figure 2 (A) shows the serine residue facing the interior of the pentameric channel. The pore is constricted in the middle, which is a largely dehydrated region, owing to the occurrence of hydrophobic residues. The kinetics of ion conduction through the pore is likely to be controlled by this long hydrophobic stretch. Initially, the nature of the pore was investigated by examining the electrostatic representation of the pore-lining residues in the channel shown in Figure 2 (B). In the surface representation, one monomer of Vpu has been omitted in the figure for clarity. The figure shows electronegative regions in red, and electropositive regions in blue. It can be seen that the top and middle region of the channel is constricted and lined entirely by nonpolar residues. Further down the channel, towards the C-terminal end, the pore widens, and the occurrence of a serine residue gives rise to a slightly hydrophilic region. The widening of the pore was shown to be due to the kink around the Ile17 residue (see Figure S1 (B)) [24], which is in agreement with experimental data [19]. The serine residue can be seen as a red region at the bottom of the channel. The occurrence of such a hydrophilic region is expected to stabilize the permeating ion, thereby assisting ion transport. It follows that the length of the hydrophobic stretch would have been greater in the absence of this serine residue. The serine therefore reduces the effective length of the hydrophobic stretch, and notably, it has been reported previously that the Ser23→Leu mutant of Vpu is inactive towards ion permeation [28].

It is not practical to model events that occur at time scales longer than the ones that are practical with MD simulations. Additionally, insufficient sampling issues preclude calculation of free energies associated with such rare events. Use of umbrella sampling, which applies a harmonic potential along the predetermined reaction coordinate, allows sampling of intermediate states in the process of ion permeation with relatively less difficulty and with significantly less computing time, while at the same time allowing the calculation of the free energy change along the reaction coordinate using methods like WHAM [57]–[59]. An important step in employing umbrella sampling is the selection of the reaction coordinate. The axis of the pore has proved to be a valuable reaction coordinate in the study of ion conduction through channels [46]–[49], [64], [65]. Using the pore axis as the reaction coordinate, the present study illustrates the energetics and atomistic mechanism of the ion channel activity of Vpu.

### Free energy profiles indicate weak ion conduction

**Figure 3:**
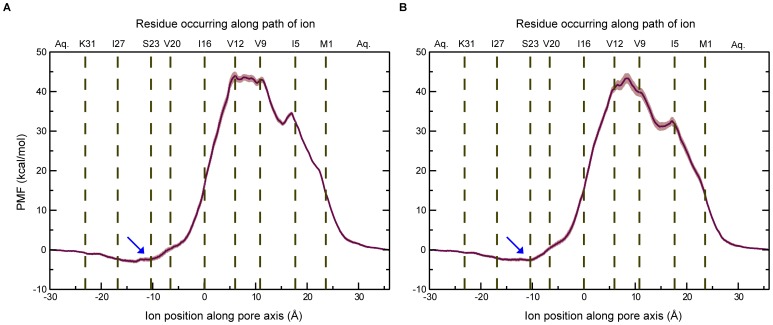
The potential of mean force (PMF) for the transport of permeating ions along the channel axis. (A) Na^+^; (B) K^+^. Residues facing the pore have been shown at their appropriate positions. The coordinates of these residues were calculated by determining the center of mass of their side chains. Error bars are shown as bands. The soft minimum around Ser23 is marked with an arrow.

The PMFs for the transport of Na^+^ and K^+^ ions are shown in Figure 3. The PMF along the pore axis is shown, with the C-terminus (or the intracellular side) on the left and the N-terminus (extracellular side) on the right. The residues lining the pore are also marked appropriately in the plots. The free energy profiles presented correspond to the ion permeating from the bulk water region outside the C-terminal end of the channel to the bulk water region on the other end of the channel. For determining the errors, the sampling data of last 1 ns/window was divided into five parts with 200 ps/window, and the PMF was computed separately for each part. The mean, standard deviation, and standard error were then calculated from the PMF obtained for the different intervals. The energy barriers for this process are about 43.9 kcal/mol for Na^+^ and 43.3 kcal/mol for K^+^. Such a high energy barrier is to be expected, given the highly hydrophobic environment that prevails inside the channel. The energy barrier observed is considerably higher than that for other known ion channels [46]–[49], [64], [65], and it suggests that the ion channel activity of Vpu is weaker than that of typical ion channels. This is in good agreement with conductance experiments suggesting that Vpu is expected to behave as a weakly conducting ion channel [28]. The high energy barrier encountered by the permeating ion is likely to make the process kinetically slow.

**Figure 4:**
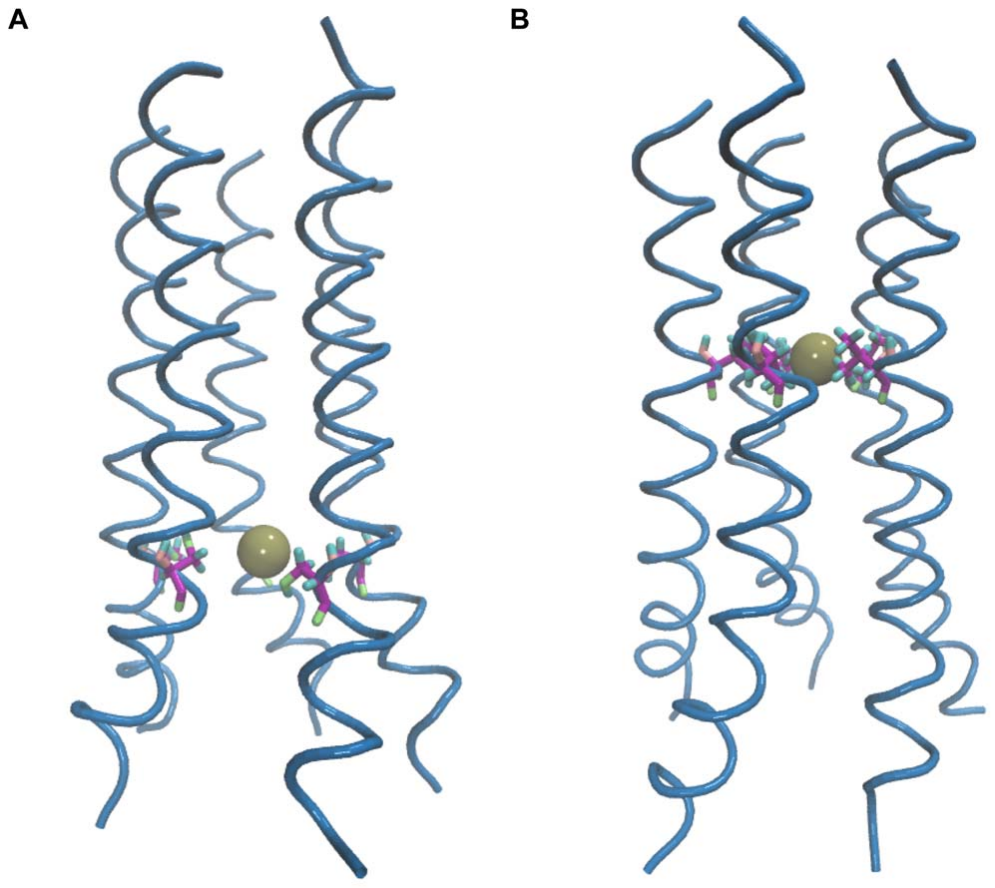
Conformations with the permeating ion inside the channel. Snapshots of the Na^+^ ion near the ring of (A) Ser23 residues and (B) Val12 residues, respectively.

The features of the two free energy profiles (Na^+^ and K^+^) were found to be very similar to each other. The movement of the ion into the channel from the C-terminal side is accompanied by the release of 2 kcal/mol of energy, with a shallow minimum occurring around *z* = −10 Å (the region is marked with an arrow in Figure 3). This corresponds to the position of the ring of Ser23 residues, and suggests the role of serine as a weak binding site. This is consistent with previous conductance studies on mutant forms of Vpu, which have proposed that the serine can act as a weak binding site for ions, since replacing the serine with a hydrophobic residue results in loss of ion channel activity. The fact that the minimum seen around the serine is shallow rather than deep indicates that the binding site is at best only a weak one. As the ion moves further into the channel, there is a surge in the free energies brought about by the unfavorable interactions that the positive ions have with the hydrophobic environment of the channel. The maximum is reached around two valine residues, namely Val9 and Val12, which occur in the middle of the hydrophobic stretch in the channel. The ions experience a soft minimum around 15 Å, which arises from the stabilization of the ion at this position by coordinating water molecules (see later). Figure 4 shows snapshots of the Na^+^ ion at two places inside the channel. The first snapshot shows the ion near the ring of Ser23 residues, while the second snapshot shows the ion near the ring of Val12 residues. As can be seen in the figure, the latter corresponds to the narrow stretch of the channel, where the energy barrier reaches a maximum.

**Figure 5:**
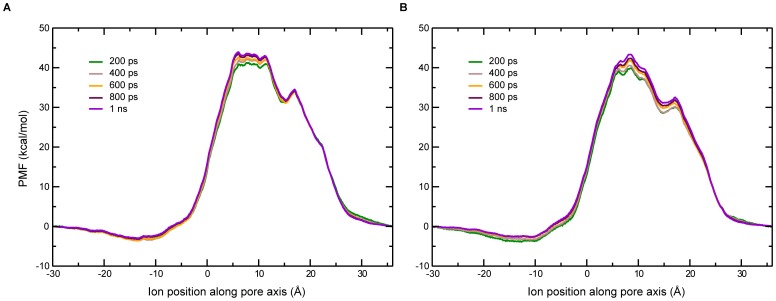
The PMF for ion transport for different sampling time periods. (A) Na^+^ (B) K^+^.

The remarkably similar free energy profiles corresponding to the permeation of Na^+^ and K^+^ explain why the channel does not discriminate Na^+^ over K^+^, as reported previously based on experimental studies [22]. Given that the windows are close to each other, 1.1 ns long MD simulations for each of the 133 windows per free energy profile is deemed adequate. The total simulation time for each of the two ions was therefore (1.1 ns * 133)≈146 ns. The PMFs obtained at different durations of the simulations and for the whole duration are given in Figure 5. The PMFs calculated agree well with each other with only minor variations in the free energy values. Based on this, modeling of the multi-ion permeation was also modeled using a similar protocol (see below).

### Extent of solvation governs ion transport kinetics

**Figure 6:**
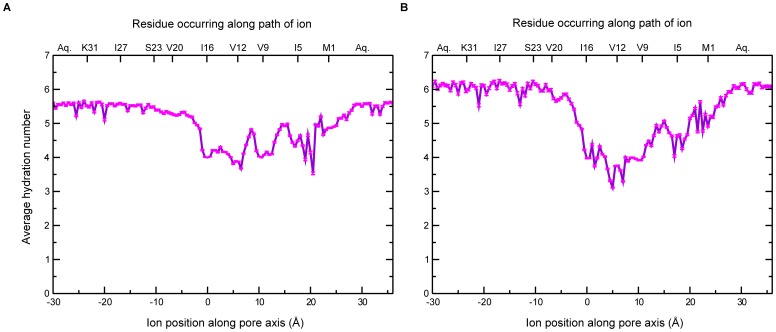
Average hydration number for permeating ions along the channel axis. (A) Na^+^; (B) K^+^. The hydration number was calculated by determining the number of water molecules within a certain cutoff distance of the permeating ion. The cutoff used was 2.8 Å for Na^+^ and 3.2 Å for K^+^. The hydration number values shown here have been averaged over the trajectory for the respective window. Error bars are shown in pink.

Solvation of the ion during the permeation process and its relationship with the free energy profiles were examined based on the hydration number, the number of water molecules in the first solvation shell of the ion and the interaction energies (see below). Figure 6 shows the average hydration number of the ions with respect to the position along the channel. The hydration numbers were calculated based on cutoff values of the distance between the ion and the oxygen atom of water molecules (2.8 Å for Na^+^ and 3.2 Å for K^+^). These cutoff values were chosen accounting for the size of the respective ions [49]. As the ion enters the C-terminal side of the channel, it retains its hydration shell, and proceeds spontaneously into the channel. The hydrophilic environment in this region is created by the polar Ser23 sidechain (this corresponds roughly to the position −10 Å in the figure). As the ion proceeds towards the N-terminal side, it gradually loses waters of hydration, with the hydration number reaching a minimum in the middle region of the channel. The loss in hydration number leads to a destabilization of the ion in the middle region relative to the C-terminal region, which is reflected in the rise in PMF. This is consistent with the earlier discussion on the nature of the pore in this region that this region is rich in hydrophobic residues, which results in stripping of water molecules from the ion and drying of the whole region in general. It must be noted that there are relatively more number of water molecules around the ion when the ion is in the region between Ile5 and Val9 (corresponding to *z* = 15 Å). This is explained by a slight widening of the pore around this region [24], making it possible to accommodate more water molecules. The stabilization of the ion by this solvation explains the occurrence of the soft minimum seen in the PMF at this position (Figure 3). As the ion proceeds towards bulk water beyond the N-terminal end of the pore, the hydration number of the ion increases, and is associated with a sharp fall in the PMF. Figure S2 shows the radial distribution function of water molecules around the permeating ion. The first hydration shell is seen as a thick band in the figure. It can be seen that although there are some water molecules beyond the first hydration shell, there is no distinct second hydration shell.

**Figure 7:**
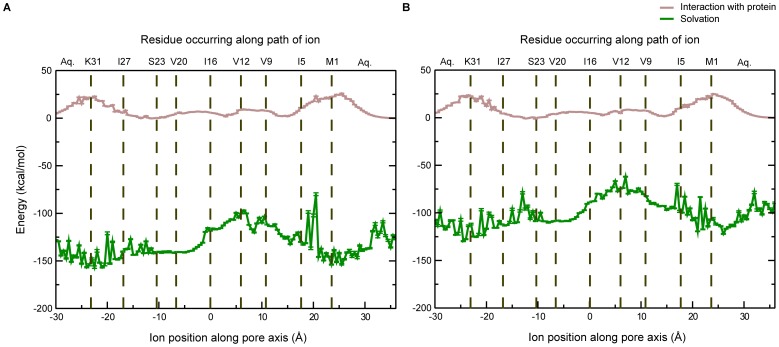
The average ion-protein interaction energy and average solvation energy for different positions of the permeating ion. (A) Na^+^; (B) K^+^. Values for each window have been averaged over the trajectory for the respective window, and are shown with error bars. The pore-lining residues are also shown at their respective position in the plot.

The permeating ion encounters two principal interacting partners inside the channel – the pore water molecules and the channel itself, and such interactions are expected to affect the free energy profiles significantly. Accordingly, interaction energies, which include the contributions of the electrostatic and Lennard-Jones (LJ) terms of the potential energy function, were calculated to examine the solvation energy. Mean values of the interaction energies between ion and water, and ion and protein are shown in Figure 7. As can be seen clearly, the major contribution comes from the solvating water molecules, whose contribution towards ion stability is several orders of magnitude higher than that offered by the protein. It is to be noted that such interaction energy calculations have limited applicability, since these are calculated based on the potential energy equation only, and do not include entropy effects, for instance. The loss of solvation in the middle region, which is seen in the figure as an increase in the solvation energy, also explains the rise in PMF in this region. The energy barrier that is seen in the free energy profile therefore arises from the desolvation of the ion. This energy barrier, in turn, is likely to make the process of ion conduction kinetically slow, making Vpu a weakly conducting ion channel.

Previous biophysical studies have suggested that Ser23 might act as a weak binding site for ions [28]. The interaction of the ion with the protein shown in Figure 7 reveals a shallow minimum for both Na^+^ and K^+^ near the serine residue, suggesting weak binding ability. On the other hand, there is a remarkable enhancement of stability due to solvation as the ion moves towards the serine; this is seen as a fall in the solvation energy of the permeating ion. The serine residue therefore enhances ion movement into the channel by giving rise to a sufficiently solvated region on the C-terminal side. There are thus two ways in which the Ser23 makes conditions conducive for ion transport: firstly, it acts as a weak binding site, and, secondly, it gives rise to a hydrophilic region around the entrance of the pore.

### Absence of concerted ion transport explains the slow kinetics

**Figure 8:**
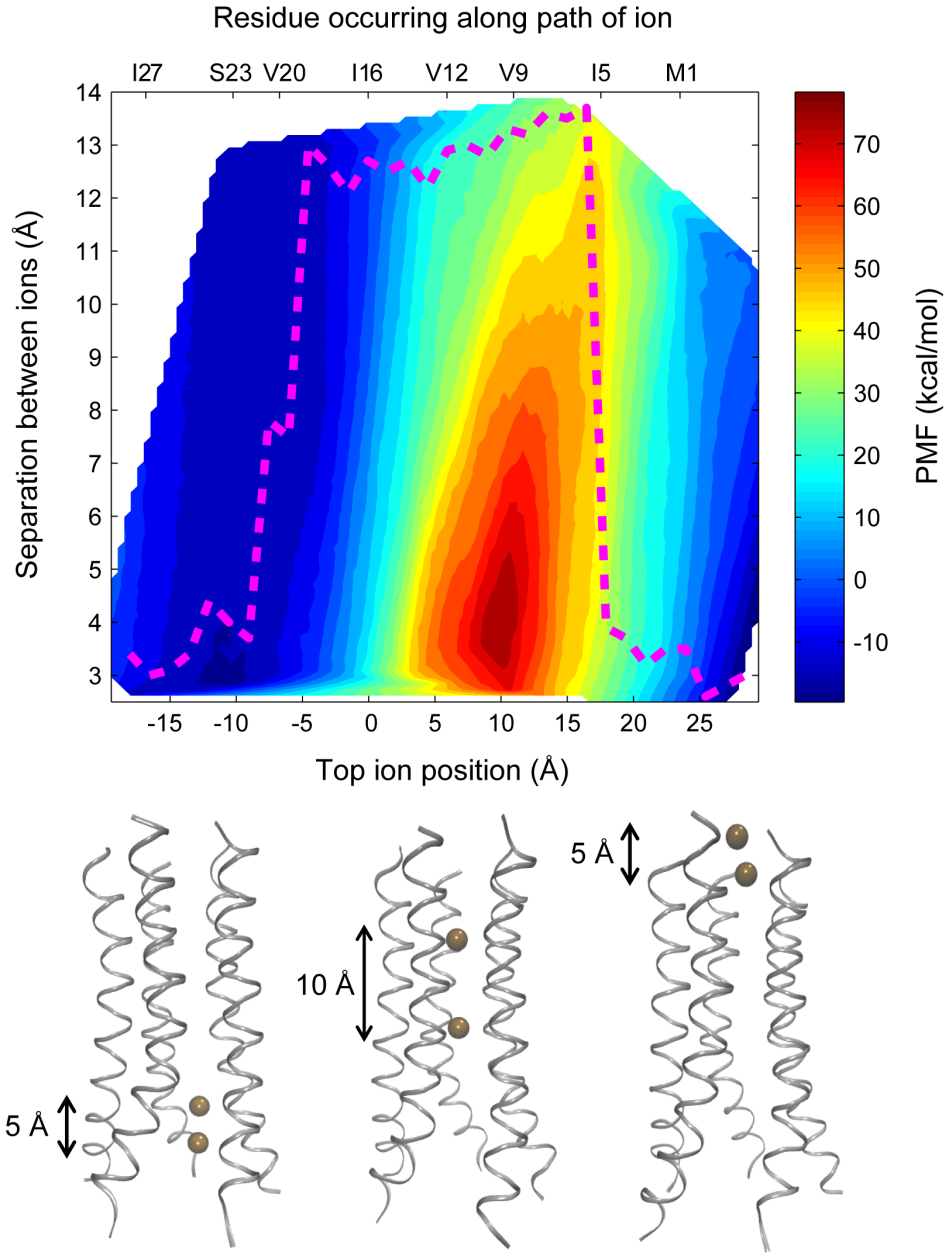
The PMF shown as a function of the positions of the two permeating ions. The x-axis labels at the bottom show the position of the top ion along the channel axis, while the labels at the top show the pore-lining residues at their respective positions along the channel axis. The pathway with the lowest free energy barrier is highlighted in maroon color. Images of ion positions corresponding to the pathway with the lowest free energy barrier are shown below the plot, together with the distance between the two ions at these positions.

To investigate if more than one ion can pass through the channel at a time, the free energy profile was calculated as a function of the positions of two permeating K^+^ ions. The free energy landscape with respect to the position along the z-axis of the first ion and the distance of the second ion from the first is shown in Figure 8. The probability distributions obtained from the biased simulations were checked for adequate sampling along both the reaction coordinates prior to construction of the free energy surface. The lowest energy pathway with respect to the position of the first ion is also marked in the plot. The structures presented below the plot show the conformations of the channel corresponding to this path at three different positions. The free energy surface as a whole shows that permeation of two ions at the same time is not thermodynamically favorable compared to the movement of one ion. If one follows the minimum energy path given in the figure, one can see that when the two ions are near the C-terminal side of the pore (bottom left corner of the figure), the ions are close in space. This is because this region is reasonably well solvated, and interactions between the two ions are shielded. As the two ions cross the hydrophilic region around Ser23 and enter the dehydrated middle region of the channel, interionic repulsion becomes significant. The reduced number of water molecules hydrating the ions, and the dry nature of this part of the pore leads to longer distances between the two ions. This means that two ions cannot enter the middle region of the channel simultaneously, and only one ion can pass at a time. Once the first ion reaches the N-terminal region, the second ion starts crossing the hydrophobic stretch in the pore. Thus a mechanism is hypothesized in which only one ion crosses the hydrophobic region of the channel at a given time while the next ion waits near the C-terminal region, until the first one reaches the N-terminal end of the pore. The kinetics of the process of ion transport is therefore seen to be controlled primarily by the hydrophobic stretch. Such a hypothesis is also consistent with previous experimental studies. Fischer and coworkers have proposed that the conductivity of Vpu follows Michaelis-Menten behavior upon increasing the salt concentration [28]. Such behavior is explained by the fact that a single ion can pass through the channel at a time, as suggested by the mechanism discussed here.

It may be noted that, although the windows for the two ion permeation were chosen such that the spacing between the two ions varied from 4 Å to 12 Å, the conformations sampled during the simulations cover a range of 3 Å to 14 Å. While conformations with the spacing close to 3 Å are sampled in the terminal regions of the channel, conformations with the spacing increasing to 14 Å are seen in the middle region of the channel. This suggests a propensity of the two ions to stay close together while they are near the hydrated terminal regions of the channel. On the other hand, in the middle region of the pore, the ions tend to stay as far apart as possible, thereby minimizing interionic repulsion.

### Perspectives

The high energy barrier reported here for the transport of K^+^ and Na^+^ through the Vpu channel can be attributed to the long stretch of hydrophobic residues which constrict the pore, and cause the dehydration of the pore lumen (Figure 1B). It might be argued that the use of restraints on the backbone C_α_ atoms of the protein (to prevent drifting) during the umbrella sampling simulations might hinder relaxation of the protein, and thereby cause the PMF to rise. However, it must be mentioned that the protein structure used here was previously subjected to unrestrained equilibration simulations in explicit solvent and bilayer for 30 ns, which is expected to be long enough for the protein backbone and sidechain atoms to relax. Furthermore, unlike transporters, ion channels do not usually undergo large scale domain motions during the process of solute transport, since they undergo gating prior to solute transport [66]. Thus the use of weak restraints on C_α_ atoms during the umbrella sampling is not likely to affect the dynamics in a significant way.

An estimate of the rate of ion conduction can be made using the Arrhenius equation. An energy barrier of around 43 kcal/mol that is reported here suggests that the rate for the ion conduction to be several orders of magnitude lower than that estimated using the conductivity measurements previously reported by Mehnert et al. [28]. Thus, although the present study is able to qualitatively account for the weak channel activity of Vpu, it is not able to quantitatively reproduce the conductance reported in experiments. The difficulty in accurately reproducing experimentally determined conductances from the height of energy barriers derived from PMFs has been reported by Sansom and coworkers [65]. One factor that could lead to inaccuracies in the PMF is the non-polarizable nature of the force field used [47], [48]. Apart from the protein, the polarizability of the water model is also greatly important, since the pore water molecules play a crucial role in stabilizing the ion inside the pore [47].

Another factor that could affect the quantification of properties of the channel is an inadequate representation (in simulations) of the conditions prevailing in electrophysiological experiments. Experimental channel recordings investigating selectivity in Vpu have been carried out under the application of a voltage [17], [21], [22], [27], [28]. It is likely that the presence of an electric potential brings about conformational changes in the channel, leading to different conductance states of the channel. A possible approach for obtaining insights into these conductance states is to model the channel in the presence of a voltage. However, Vpu has been shown to exhibit a variety of conductance states even if a very small potential is applied [27]. Furthermore, the open time for the channel has been shown to be voltage-independent [27]. The factors leading to the occurrence of the different conductance states, therefore, are still not properly understood. It has been suggested that gating in the channel might be regulated by the lateral membrane pressure and the lipid composition [27]. Thus, an understanding of the different conductance states might be possible by modeling ion permeation through the channel in a number of phospholipid environments with varying lateral pressure. Further studies are necessary in understanding the role of applied external voltage on the structure and dynamics of Vpu, and the resultant changes in ion permeation thermodynamics/mechanism if any.

### Conclusions

Free energy profiles for conduction of monovalent cations across the Vpu channel have been characterized using umbrella sampling free energy calculations. The calculations reveal reasonably high energy barriers for ion movement through the channel, owing to the highly hydrophobic environment of the pore. The high energy barriers suggest that ion transport across the pore is kinetically slow, making Vpu a weakly conducting channel. The weak ion conducting activity is consistent with experimental biophysical studies, and has been explained based on the free energy calculations and hydration properties. The energy barriers arise from the desolvation of the ion as it moves from bulk water to the middle hydrophobic region of the channel. The permeation of Na^+^ and K^+^ involves comparable energetic costs, explaining why the channel does not discriminate Na^+^ over K^+^. The results show that the Ser23 residue plays a role in ion channel activity by giving rise to a hydrophilic region in the pore, and by serving as a weak binding site. The possibility of multi-ion permeation has been investigated by computing the free energy as a function of the positions of two permeating ions. Results show that only one ion can pass at a time through the middle hydrophobic region of the channel. The mechanism proposed is able to account for the slow kinetics of ion transport across the channel, and it suggests a role for the hydrophobic stretch in controlling the kinetics of the process.

## Supporting Information

Figure S1 **Orientation of important residues in the channel.** (A) Glu28, Tyr29, Arg30, and Lys31 residues. Arg30 and Tyr29 are seen interacting with nearby lipid headgroups. Arg30 also forms a salt bridge with Glu28 on an adjacent helix. Lys31 faces the pore, but is very unlikely to exert any influence on the permeating ion, since it is shielded by a large number of water molecules. (B) Kink in the helix around the Ile17 residue.
(TIF)

Figure S2 **Radial distribution function for water molecules around the permeating ion.** (A) Na^+^ (B) K^+^.
(TIF)
