# Supplementary figures and images for: Atomistic Detailed Mechanism and Weak Cation-Conducting Activity of HIV-1 Vpu Revealed by Free Energy Calculations
Source: PLoS One. 2014 Nov 13;9(11):e112983. doi: 10.1371/journal.pone.0112983 (PMC4231112; doi:10.1371/journal.pone.0112983)

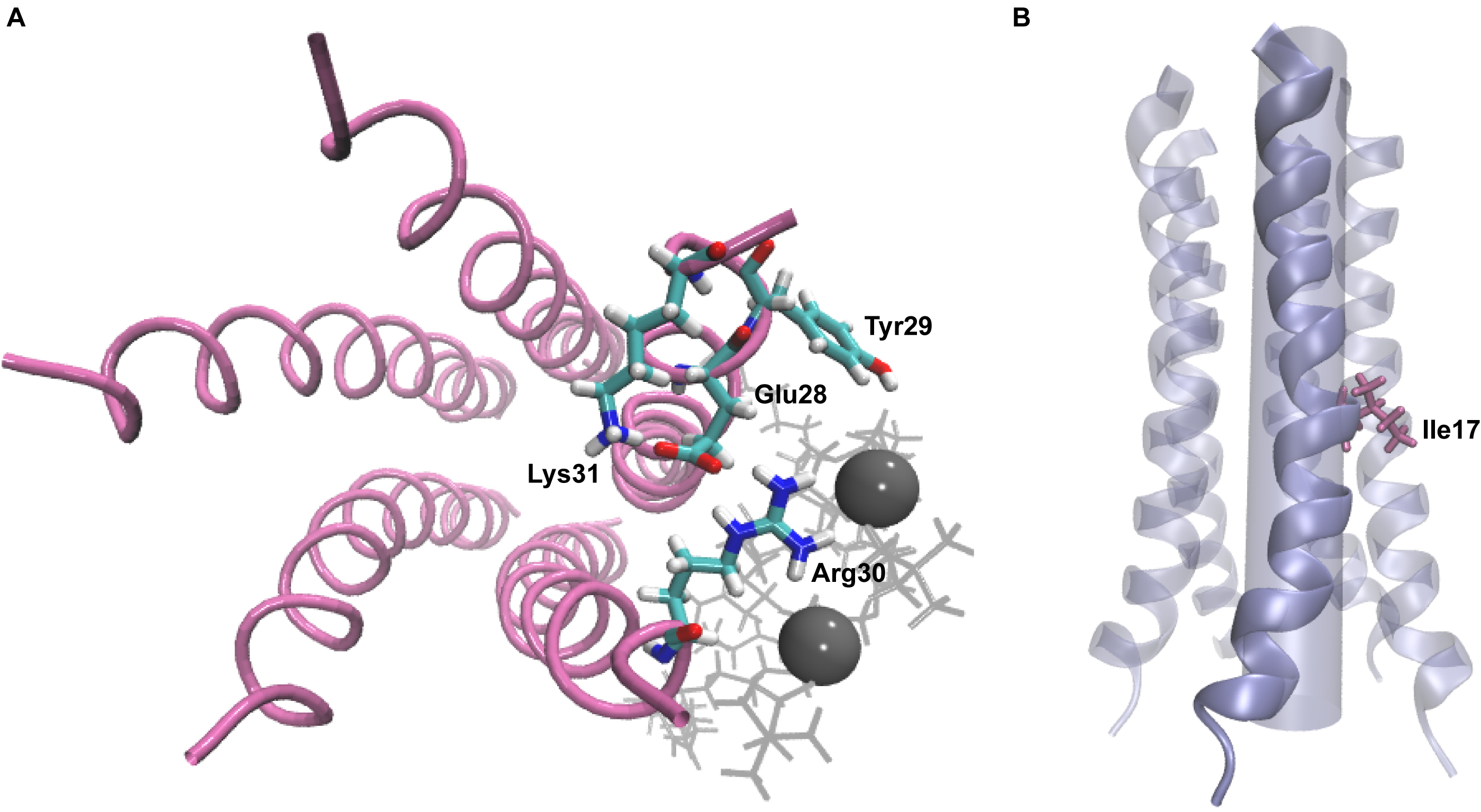

Supplement: Figure S1 — Orientation of important residues in the channel. (A) Glu28, Tyr29, Arg30, and Lys31 residues. Arg30 and Tyr29 are seen interacting with nearby lipid headgroups. Arg30 also forms a salt bridge with Glu28 on an adjacent helix. Lys31 faces the pore, but is very unlikely to exert any influence on the permeating ion, since it is shielded by a large number of water molecules. (B) Kink in the helix around the Ile17 residue. (TIF) [file pone.0112983.s001.tif]

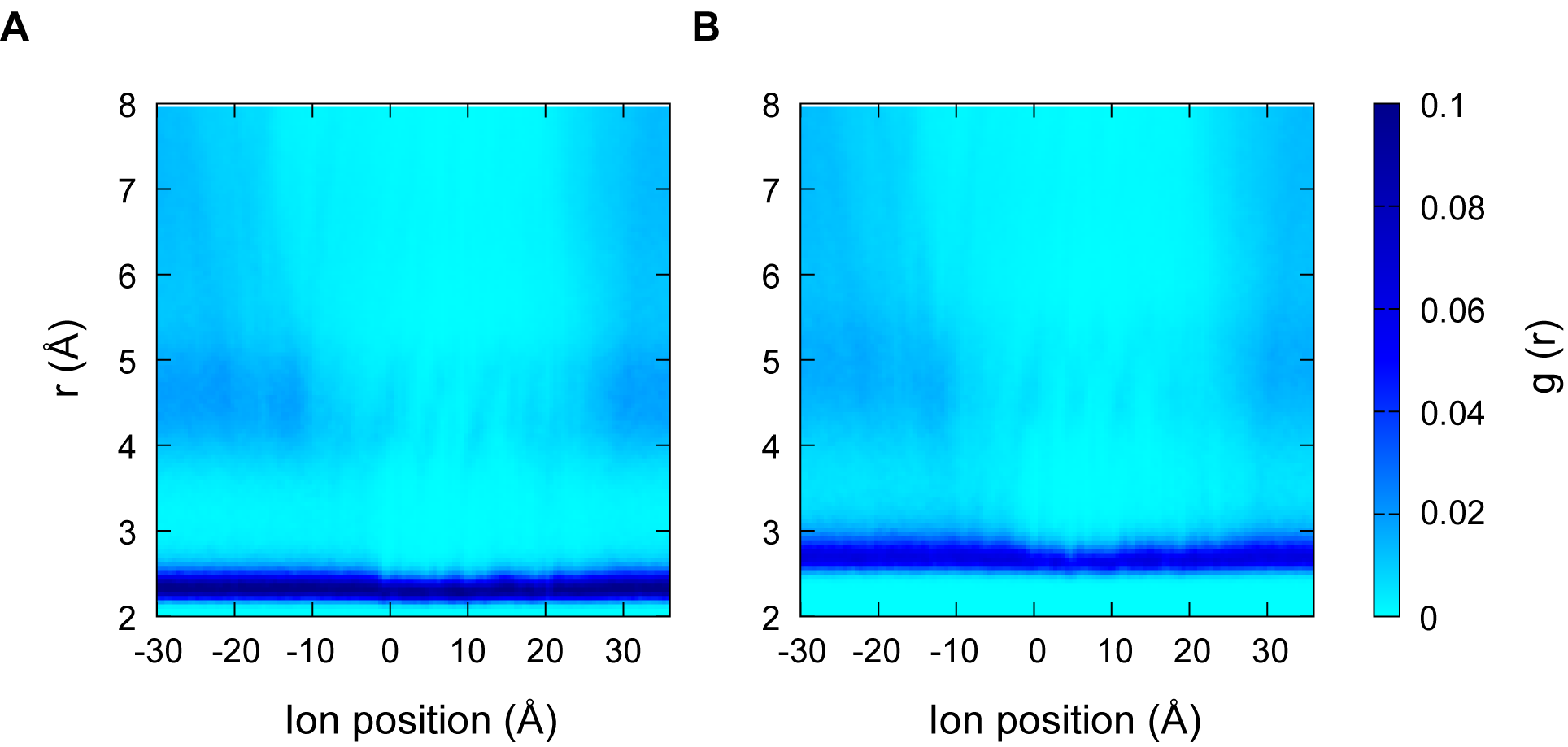

Supplement: Figure S2 — Radial distribution function for water molecules around the permeating ion. (A) Na+ (B) K+. (TIF) [file pone.0112983.s002.tif]
